# Extract from a Notice of Recent Researches on the Origin of Entozoa, More Especially of Tape-Worms

**Published:** 1856-11

**Authors:** Allen Thompson

**Affiliations:** London and Edinburgh, and Professor of Anatomy in the University of Glasgow


*Extract from a notice of Recent Researches on the Origin of Entozoa, more especially of Tape Worms.* By ALLEN THOMPSON, M.D., F.R.S., London and Edinburgh, and Professor of Anatomy in the University of Glasgow.

There can be no doubt, whatever, that the occurance of tape-worm in the human subject, as in animals, is dependent on the introduction into the alimentary canal of the Scolex-larva, accidentally or along with food. The most frequent though not the only, source of these Scolices in this country and a part of the continent of Europe, is probably the Cysticercus cellulosæ of measly pork, when this is used in a partially cooked or raw state. This accords with general belief, and with what has been ascertained in a number of instances of persons affected with tape-worm, viz.: that they had been in the habit of eating raw or imperfectly-cooked meat. In Abyssini, where this habit prevails to a great extent, the inhabitants are well known to be remarkably subject to tape-worm; indeed, in that country the affection is looked upon as entirely a natural one

The difference in the prevalence of Tænia solium in this country and in western Europe, and of the Bothriocephalus latus in the eastern division of the Continent, is well known; but I am not aware whether any observations have yet been made upon the most probable source of the latter entozoon. In Russia, however, where the Bothriocephalus is the usual tape-worm, it has been found that the long continued use of an exclusive animal diet, such as has been recommended for the cure of some diseases, has been followed by the occurrence of Tænia solium. In Switzerland, also, in the eastern parts of which the Bothriocephalus prevails, it has been observed that the hogs are rarely, if ever effected with Cysticercus; but occasionally pork is introduced from France strongly tainted with this affection, and this may account for the occasional occurence of the Tænia solium, especially in Western Switzerland.*

These circumstances seem to point out very clearly the means to be adopted for the prevention of this troublesome complaint. At the same time, it is probable that there may be some accidental means by which these larvæ of the tape-worm may be introduced; and it will be easily understood how this may more particularly happen in the cases of butchers, cooks, or others in the habit of handling affected meat.

The instances in which the human body is affected with the Cysticercus, or other cystic entozoa, though not very rare, are by no means so frequent as the tape-worm; but they are much more serious in their effects, more obscure in their origin, and in the meantime more difficult to prevent. Scarcely any attention has yet been given to the source from which the various cystic entozoa infesting the human body may have derived their origin; but the observations already referred to make it extremely probable that the explanation of their introduction is to be sought for in the same causes which have been shown to operate in the lower animals. Thus it appears to have been demonstrated that the Cænurus of the sheep proceeds from the ova or first embryos of Tænia, and it is most probable that these are obtained from the dog. The only mode, therefore, of

* See the notice of a case, in which it appeared that the abstinence from the practice of eating raw meat during some time, effected a cure of inveterate tape-worm, with which a person had been long affected, in the June number of the *Edin. Monthly Jour. of Med.* for the present year. A gentleman of my acquaintance, who has long been affected with a very large and inveterate tape-worm, informs me, that formerly he was in the habit of eating animal food imperfectly cooked.

removing this affection from a flock in which it may have become prevalent, and in which it is well known sometimes to cause very great losses, must be the careful separation of the dog from the sheep for a certain time: for such time, indeed, as that the dog shall find no more Cænuri in the offal, &c. of the sheep, in eating which it receives the larvæ of its Tænia, and the dog being free from this Tænia, shall not furnish the ova or embroys, which being taken accidentally with the pasturage or water by the sheep, establish themselves in them as encysted Cænuri. Von Siebold states the important fact, that those flocks which are entirely without dogs, and are stall-fed, are never affected with the sturdy.

A remarkable example of the presence of cystic entozoa in the human subject, is mentioned by Von Siebold as having recently been described by Dr. Schleisner, in his "Medical Topography of Iceland," published 1851. It appears that the people of that country have been for some time suffering, to a great extent, under a very remarkable hydated disease. The Hydatids affect the liver, peritoneum, and sub-cutaneous texture. Eschricht writes to Von Siebold, that this disease has extended itself to such an alarming degree, about a sixth of the whole population being affected with it, that it is attracting considerable attention at Copenhagen. It produces long-protracted illness, and terminates in a painful death; and means of cure have not yet been discovered. Von Siebold considers it as extremely probable that this disease, consisting in the development of a cystic entozoon, depends on the introduction of the ova of a Tænia into the body; and that this arises from the immense quantity of dogs kept in Iceland for the purpose of herding sheep and cattle. Should the further elucidation of this fact lead to the adoption of successful measures for the preventation of the disease, it will be a satisfactory instance of the assistance which may be furnished to rational pathology and the practice of medicine, from physiological researches, which might at first sight have appeared to some to be very remote from such an application.

Before concluding, I would call the attention of medical practitioners, more directly than heretofore, to the investigation of the habits and circumstances of patients who may be under their care for various verminous affections. There is another department of the subject upon which I have been unable to touch, which is also greatly deserving of increased attention; I mean the collection of observations by those who may be favorably situated, as to the nature of the entozoa which affects different races and nations of mankind, together with the circumstances and modes of life which may seem to have an influence in determining the nature of the entozoa in different countries. As a single example of what may be expected from well-conducted observations of this kind, I may here mention, that, at Von Siebold's suggestion, Dr. Bilharz, being in charge of making dissections of the dead bodies in the hospital of Cairo, has already, within the short space of two years, discovered five entozoa with which the Egyptian and other native Africans are affected, and some of them very frequently and to a great extent, which are different from those which have long been known as the common entozoa of the European races.—*Glasgow Medical Journal—New Hampshire Journal of Medicine.*

